# Supplementary material for: Exercise and aerobic capacity in individuals with spinal cord injury: A systematic review with meta-analysis and meta-regression
Source: PLoS Med. 2023 Nov 27;20(11):e1004082. doi: 10.1371/journal.pmed.1004082 (PMC10712898; doi:10.1371/journal.pmed.1004082)
Supplement: S2 File — (DOCX) [file pmed.1004082.s003.docx]

**Electronic Supplementary Material 2**

**Exercise and aerobic capacity in individuals with spinal cord injury: A systematic review with meta-analysis and meta-regression**

**PLoS Medicine**

Hodgkiss, D.D^1^, Bhangu, G^2,3^, Lunny, C^4^, Jutzeler C.R^5,6^, Chiou S.Y^1,7,8,9^ Walter, M^2,10^, Lucas S.E^1,7^, Krassioukov, A.V.^2,11,12^, Nightingale, T.E.^1,2,9^*

**^1^** School of Sport, Exercise and Rehabilitation Sciences, University of Birmingham, UK.

**^2^** International Collaboration on Repair Discoveries (ICORD), University of British Columbia, Vancouver, Canada. **^3^** MD Undergraduate Program, Faculty of Medicine, University of British Columbia, Vancouver, Canada. **^4^** Knowledge Translation Program, Li Ka Shing Knowledge Institute, St. Michael’s Hospital, Toronto, and the University of British Columbia, Vancouver, Canada. **^5^** Department of Health Sciences and Technology, ETH Zurich, Zurich, Switzerland. **^6^** Schulthess Clinic, Zurich, Switzerland. **^7^** Centre for Human Brain Health, University of Birmingham, United Kingdom. **^8^** MRC Versus Arthritis Centre for Musculoskeletal Ageing Research, University of Birmingham, United Kingdom. **^9^** Centre for Trauma Science Research, University of Birmingham, United Kingdom. **^10^** Department of Urology, University Hospital Basel, University of Basel, Basel, Switzerland. **^11^** Department of Medicine, Division of Physical Medicine and Rehabilitation, University of British Columbia, Vancouver, Canada. **^12^** GF Strong Rehabilitation Centre, Vancouver Coastal Health, Vancouver, Canada.

*** Corresponding author:** Tom E. Nightingale PhD, [T.E.Nightingale@bham.ac.uk](mailto:T.E.Nightingale@bham.ac.uk)

| **S2 Table A.** Methods of exercise intensity prescription classified into thresholds provided by the ACSM [1]. | | | | | |
| --- | --- | --- | --- | --- | --- |
|  | **%V̇O_2peak_** | **%HR_peak_** | **%V̇O_2reserve_ or %HRR** | **RPE (Borg 6 – 20 scale)** | **%1 RM** |
| Light | 37 – 45 | 57 – 63 | 30 – 39 | 9 – 11 | 30 – 49 |
| Moderate | 46 – 63 | 64 – 76 | 40 – 59 | 12 – 13 | 50 – 69 |
| Vigorous | 64 – 90 | 77 – 95 | 60 – 89 | 14 – 17 | 70 – 84 |
| Supramaximal | ≥91 | ≥96 | ≥90 | ≥18 | ≥85 |
| RPE assessed via the Borg (6 – 20 scale) [2]. %1 RM, percentage of one repetition maximum; ACSM, American College of Sports Medicine; %HR_peak_, percentage of peak heart rate; %HRR, percentage of heart rate reserve; RPE, rating of perceived exertion; %V̇O_2peak_, percentage of peak oxygen uptake; %V̇O_2reserve_, percentage of oxygen uptake reserve. | | | | | |

**References**

1. American College of Sports Medicine. *ACSM’s guidelines for exercise testing and prescription.* 10^th^ edn. Baltimore, MD: Lippincott Williams and Wilkins, 2017.
2. Borg GA. Physical performance and perceived exertion. *Med Sci Sports Exerc* 1982;14:377-381.
