# Supplementary material for: Exercise and aerobic capacity in individuals with spinal cord injury: A systematic review with meta-analysis and meta-regression
Source: PLoS Med. 2023 Nov 27;20(11):e1004082. doi: 10.1371/journal.pmed.1004082 (PMC10712898; doi:10.1371/journal.pmed.1004082)
Supplement: S9 File — (1) Forest plots for each CRF outcome. (DOCX) [file pmed.1004082.s010.docx]

**Electronic Supplementary Material 9**

**Exercise and aerobic capacity in individuals with spinal cord injury: A systematic review with meta-analysis and meta-regression**

**PLoS Medicine**

Hodgkiss, D.D^1^, Bhangu, G^2,3^, Lunny, C^4^, Jutzeler C.R^5,6^, Chiou S.Y^1,7,8,9^ Walter, M^2,10^, Lucas S.E^1,7^, Krassioukov, A.V.^2,11,12^, Nightingale, T.E.^1,2,9^*

**^1^** School of Sport, Exercise and Rehabilitation Sciences, University of Birmingham, UK.

**^2^** International Collaboration on Repair Discoveries (ICORD), University of British Columbia, Vancouver, Canada. **^3^** MD Undergraduate Program, Faculty of Medicine, University of British Columbia, Vancouver, Canada. **^4^** Knowledge Translation Program, Li Ka Shing Knowledge Institute, St. Michael’s Hospital, Toronto, and the University of British Columbia, Vancouver, Canada. **^5^** Department of Health Sciences and Technology, ETH Zurich, Zurich, Switzerland. **^6^** Schulthess Clinic, Zurich, Switzerland. **^7^** Centre for Human Brain Health, University of Birmingham, United Kingdom. **^8^** MRC Versus Arthritis Centre for Musculoskeletal Ageing Research, University of Birmingham, United Kingdom. **^9^** Centre for Trauma Science Research, University of Birmingham, United Kingdom. **^10^** Department of Urology, University Hospital Basel, University of Basel, Basel, Switzerland. **^11^** Department of Medicine, Division of Physical Medicine and Rehabilitation, University of British Columbia, Vancouver, Canada. **^12^** GF Strong Rehabilitation Centre, Vancouver Coastal Health, Vancouver, Canada.

*** Corresponding author:** Tom E. Nightingale PhD, [T.E.Nightingale@bham.ac.uk](mailto:T.E.Nightingale@bham.ac.uk)

***Purpose:*** This supplementary file contains a sub-analysis of gait training cardiopulmonary exercise test (CPET) modalities. The purpose was to compare whether there are any transfer effects between a gait training intervention and upper-body exercise (i.e., arm crank ergometry performance in a CPET).

***Conclusion:*** Although there are no significant subgroup differences between arm-crank ergometry and treadmill performance following a gait training exercise intervention, there are larger pooled effect estimates for absolute and relative peak oxygen consumption in interventions using a treadmill CPET.

**S9 Fig A.** Forest plot of absolute peak oxygen consumption with gait training interventions grouped into subgroups by cardiopulmonary exercise test (CPET) modality. Subgroup difference p-value was adjusted for multiple comparisons; statistically significant at *p*<0.025.

**S9 Fig B.** Forest plot of relative peak oxygen consumption with gait training interventions grouped into subgroups by cardiopulmonary exercise test (CPET) modality. Subgroup difference p-value was adjusted for multiple comparisons; statistically significant at *p*<0.025.

**S9 Fig C.** Forest plot of peak power output with gait training interventions using arm-crank ergometry (ACE) cardiopulmonary exercise test (CPET). No interventions used treadmill CPET.
