# Supplementary material for: Exercise and aerobic capacity in individuals with spinal cord injury: A systematic review with meta-analysis and meta-regression
Source: PLoS Med. 2023 Nov 27;20(11):e1004082. doi: 10.1371/journal.pmed.1004082 (PMC10712898; doi:10.1371/journal.pmed.1004082)
Supplement: Attachment — Submitted filename: hodgkiss.pdf [file pmed.1004082.s015.pdf]

This paper reports a systematic review with meta–analysis of studies of exercise in people living with spinal cord injury.

I was asked for a statistical report and I interpret that to include all aspects of the design and conduct of the study.

Points of detail

Title Since the authors have included observational studies in their review I think the word impact might be avoided in favour of something more neutral. If the review was restricted to trials then impact would be fine.

Page 7 The authors themselves state on page 28 that they are aware that restricting the study to English language only is a limitation so why do it?

Page 8 For reproducibility we need to know which information was supplied and which we cannot find in the primary papers. Acknowledging the people who provided data would be nice too.

Page 9 In fact the correlations are labelled Table e5. These seem very high for pre–post correlations given that they should not exceed the reliability of the measure. The formulas look right though.

Page 9 The robvis package is properly cited on page 11 but the packages used for the meta–analysis does not appear to be mentioned. From the GitHub repository it seems both metafor and meta were used.

Page 10 Deciding to choose the meta–analysis model based on observed heterogeneity is often recommended but these days it is usually preferred to base it on the scientific situation. See Hedges and Vevea (1998) for an early argument and Rice et al. (2018) for a detailed modern version. To quote the latter:

> Measures of heterogeneity should not be used to determine whether fixed effects analysis is appropriate, but users should instead make this decision by deciding whether fixed effects analysis — or some variant of it — answers a question that is relevant to the scientific situation at hand.

It is up to the authors to decide which fits their scientific question best.

Page 10 What funnel plots and regression tests hope to identify is small study effects. Calling it publication bias implies you know the aetiology.

Page 15 It is hard to know what sense to make of the findings on what the authors term imputed data. Reading data off a graph should not produce a systematic bias so does this imply conversion from medians is biased?

Page 15 I think that in the moderator analysis the authors need to be more cautious. These are ecological effects so, for instance, it is not age which leads to increase in the CRF outcomes but being enrolled in a study with a higher average age. Although related these are not the same.

Page 16 I agree the funnel plots should not provoke any anxiety although in e4 and e6 the outlier spoils the visual impact. That study is probably unlikely to affect the fit of the regression line given its position although it is potentially a high leverage point.

Page 17 Most people would hold that RCTs provide a better assessment of the effect of interventions so I wonder why the analysis of RCTs is treated as a sensitivity analysis rather than as the main analysis?

Page 21 Saying the first is a hostage to fortune, 'to our knowledge' perhaps safer.

Points of more substance

Subgroups

The subgroup analysis in Tables 3 and 4 includes categories for mixed and not reported. This seems to me hard to justify. Mixed is a mixture in unknown proportions of the two clearly defined categories and not reported may be a mixture or it may be purely one category. I suggest that for the subgroups the analysis is restricted to just those which can be unambiguously allocated.

Multiplicity

We can see in various places, for instance Figures e1 to e3 in supplementary material S4, that some studies are repeated. This violates one of the assumptions of meta–analysis that the effect sizes are independent.

Language restriction

Various empirical studies have indicated that the main bibliographic databases have varied and incomplete coverage of material not in English (Pilkington

et al., 2005; Shenderovich et al., 2016), and have limited coverage of material from low and middle–income country journals (Kieling et al., 2009; Syed Sheriff et al., 2008). Despite that the authors' search does appear to have turned up some relevant studies so extending to databases with coverage of other literatures might be helpful. WHO sponsors some. In the event of all the authors being monoglot English speakers they might be interested in an empirical study of machine translation in reviews (Jackson et al., 2019). There has been a study in this journal which went to extraordinary lengths to obtain full world-wide coverage (James et al., 2022)

Summary

This is an extremely detailed report of a substantial review and the length of my comments reflects that.

Michael Dewey

References

L V Hedges and J L Vevea. Fixed– and random–effects models in meta–analysis. *Psychological Methods*, 3:486–504, 1998.

J L Jackson, A Kuriyama, A Anton, A Choi, J-P Fournier, A-K Geier, F Jacquerioz, D Kogan, and R Sun. The accuracy of Google Translate for abstracting data from non–English–language trials for systematic reviews. *Annals of Internal Medicine*, 171:677–679, 2019.

T James, N Mukadam, A Sommerlad, H R Pour, M Knowles, I Azocar, and G Livingston. Protection against discrimination in national dementia guideline recommendations: A systematic review. *PLOS Medicine*, 19 (e1003860), 2022.

C Kieling, H Herrman, V Patel, and J J Mari. Indexation of psychiatric journals from low– and middle–income countries: a survey and a case study. *World Psychiatry*, 8:40–44, 2009.

K Pilkington, A Boshnakova, M Clarke, and J Richardson. 'No language restrictions' in database searches: what does this really mean? *The Journal of Alternative and Complementary Medicine*, 11:205–207, 2005.

K Rice, J P T Higgins, and T Lumley. A re–evaluation of fixed effect(s) meta–analysis. *Journal of the Royal Statistical Society*, 181:205–227, 2018.

Y Shenderovich, M Eisner, C Mikton, F Gardner, J Liu, and J Murray. Methods for conducting systematic reviews of risk factors in low– and middle–income countries. *BMC Medical Research Methodology*, 16(32), 2016.

R J Syed Sheriff, C E Adams, P Tharyan, M Jayaram, L Duley, and the PRACTIHC Mental Health Group. Randomised trials relevant to mental health conducted in low and middle–income countries: a survey. *BMC Psychiatry*, 8(69), 2008.
