# Supplementary material for: Exercise and aerobic capacity in individuals with spinal cord injury: A systematic review with meta-analysis and meta-regression
Source: PLoS Med. 2023 Nov 27;20(11):e1004082. doi: 10.1371/journal.pmed.1004082 (PMC10712898; doi:10.1371/journal.pmed.1004082)
Supplement: Attachment — Submitted filename: PLOS Medicine Paper Review - 08 March 2023.docx [file pmed.1004082.s016.docx]

Number: PMEDICINE-D-22-02587R1

Title: The impact of physical activity and exercise on aerobic capacity in individuals with spinal cord injury: A systematic review with meta-analysis and meta-regression.

Keywords: cardiorespiratory fitness, cardiopulmonary fitness, function, spinal cord injuries, rehabilitation, exercise

- This manuscript will represent a valuable addition to the literature. It represents a carefully conducted systematic review to determine the effect of exercise interventions and SCI characteristics alter cardiorespiratory fitness, defined by change in absolute VO2peak, relative VO2peak, and/or peak power output.
- There were multiple important research questions that were addressed in this work. Congratulations to the authors for developing this comprehensive review.
- This review will be a strong contribution to the literature as it stands. However, according to the Cochrane guidelines, it is recommended that the authors rerun or update the search within 12 months prior to publication.

Minor Revisions:

- Line 74-76: suggestion to revise the statement to include what there is a significant improvement from? For example, add to the end of the statement: “in all 90 studies after an exercise intervention”.
- Line 86-88: Great summary statement. It is unclear why this final conclusion statement only focuses on power output
- Line 105: Consider adding the word “factors” between “confounding and”.
- Line 130: Consider removing the second mention of “cardiorespiratory fitness” and replace with CRF. The acronym was mentioned in Line 129.
- Line 176: Consider revising to “…and not other *outcomes* in individuals with SCI.”
- Line 430: Were adverse events defined as events causing cessation of exercise or reported after the exercise intervention? Consider defining as serious adverse events for events that caused cessation of the exercise session or exercise intervention and adverse events for those that were reported after the session or intervention.
- Line 435-440: These quotes are helpful to inform the nature of the adverse events. Are the quotes presented the most extreme adverse events? Consider describing how these quotes were chosen for Section 3.1.3.
- Line 584-585: Consider revising to clarify this statement. Are the authors suggesting that exercise intervention studies conducted in the inpatient rehabilitation phase are providing concurrent rehabilitation (i.e., a greater dose of exercise per week due to study involvement)?
- Line 602-603: It is unclear why there is a W after 16 and 9 in this sentence.
- Line 715-617: This section on exercise intensity is thoughtfully written. Consider adding an additional sentence to provide further direction on how to corroborate safety and feasibility of high intensity interval training (HIIT). Alternatively, perhaps there is a recommendation for the style of HIIT protocol to prescribe.
