# Supplementary material for: Exercise and aerobic capacity in individuals with spinal cord injury: A systematic review with meta-analysis and meta-regression
Source: PLoS Med. 2023 Nov 27;20(11):e1004082. doi: 10.1371/journal.pmed.1004082 (PMC10712898; doi:10.1371/journal.pmed.1004082)
Supplement: Attachment — Submitted filename: Rebuttals_Hodgkiss-et-al.docx [file pmed.1004082.s017.docx]

We thank the editors and the reviewers for their positive comments and suggestions. We have addressed their questions and comments point-by-point below.

**Editorial Comments**

**1.**	**Title:** Please revise your title according to PLOS Medicine's style. Your title must be nondeclarative and not a question. It should begin with main concept if possible. "Effect of" should be used only if causality can be inferred, i.e., for an RCT. Please place the study design ("A randomized controlled trial," "A retrospective study," "A modelling study," etc.) in the subtitle (ie, after a colon).

**Response:** We have edited our title to ‘‘Effect of exercise on aerobic capacity in individuals with spinal cord injury: A systematic review with meta-analysis and meta-regression’, in accordance with your guidance. Given that we have now restructured the manuscript to include only RCTs in the primary meta-analysis, we believe that the use of “Effect of” is justified. In keeping with this change, given the RCTs have primarily tested ‘exercise’ rather than ‘physical activity’ interventions, we have chosen to remove ‘physical activity’ from the title and in other areas of the manuscript where appropriate. In keeping with this change, given the RCTs have primarily tested ‘exercise’ rather than ‘physical activity’ interventions, we have chosen to remove ‘physical activity’ from the title and in other areas of the manuscript where appropriate.

**2.**	**Abstract:** Please report your abstract according to PRISMA for abstracts, following the PLOS Medicine abstract structure (Background, Methods and Findings, Conclusions) <http://www.plosmedicine.org/article/info:doi/10.1371/journal.pmed.1001419>

**Response:** Our abstract is now reported according to PRISMA and the PLOS Medicine abstract structure.

**3.**	**Abstract:** Please provide the data sources, number of studies included, types of study designs included, and synthesis/appraisal methods.

**Response:** Thank you for this comment. With a view to keeping the abstract concise, we initially only provided details of the primary meta-analysis from our manuscript (e.g., pre-post and RCT studies combined). Given the more flexible abstract word limit for PLOS Medicine we have now edited the abstract to include information on studies included with other study designs (e.g., cohort, cross-sectional and observational), which were included as secondary meta-analyses in our manuscript. We have now also provided information on the databases that were searched in the abstract.

**4.**	**Abstract:** Abstract Background: Provide the context of why the study is important. The final sentence should clearly state the study question.

**Response:** Our background now provides additional context on the importance of the study, highlighting that a low CRF is a leading risk factor for chronic disease and that this may be modified by physical activity and exercise.

**5.**	**Abstract:** Please ensure that all numbers presented in the abstract are present and identical to numbers presented in the main manuscript text.

**Response:** We are confident that the abstract presents the same numbers as in the main manuscript text.

**6.**	**Abstract:** In the last sentence of the Abstract Methods and Findings section, please describe the main limitation(s) of the study's methodology.

**Response:** Thank you for this comment. We have now included the main limitation of our review.

**7.**	**Author Summary:** Please delete the ‘Key Points’ and replace them with a short, non-technical Author Summary of your research to make findings accessible to a wide audience that includes both scientists and non-scientists. The Author Summary should immediately follow the Abstract in your revised manuscript. This text is subject to editorial change and should be distinct from the scientific abstract. Please see our author guidelines for more information: <https://journals.plos.org/plosmedicine/s/revising-your-manuscript#loc-author-summary>

**Response:** We have now included a non-technical author summary in place of our key points.

**8.**	**PRISMA Checklist:** Please provide the completed PRISMA checklist, provided at <http://www.equator-network.org/reporting-guidelines/prisma/>. When completing the checklist, please use section and paragraph numbers, rather than page numbers.

**Response:** This has been included as “S1 Checklist”.

**9.**	**PRISMA:** Please add the following statement, or similar, to the Methods: "This study is reported as per the Preferred Reporting Items for Systematic Reviews and Meta-Analyses (PRISMA) guideline (S1 Checklist)."

**Response:** We have added this line to the methods section (Line 235) and have provided the checklist as supplementary material.

**10.** **Updated search:** Please update your search to the present time.

**Response:** The search has been updated to include studies published between 18th July 2021 and 25th March 2023. An additional 8 studies have been included as a result.

**11.** **English language sources:** Please include non-English language sources of studies.

**Response:** We have re-screened the texts that were originally excluded as a result of using non-English language using Google translate. We can confirm that three studies met the inclusion criteria and have therefore been included. Other non-English language sources remained excluded as a result of other exclusion criteria, with the PRISMA flow chart updated accordingly. In our updated search there was no restriction on language either. We are confident that this updated approach is inclusive of all published research on this topic.

**12.** **Discussion:** Please present and organize the Discussion as follows: a short, clear summary of the article's findings; what the study adds to existing research and where and why the results may differ from previous research; strengths and limitations of the study; implications and next steps for research, clinical practice, and/or public policy; one-paragraph conclusion.

**Response:** The discussion is organised as requested. The first paragraph provided a concise summary of the article’s findings. The subsequent paragraphs discuss how the findings for each injury characteristic and exercise intervention parameter adds or differs to existing literature. We then evaluate the strengths and limitations of our study, as well as discuss the limitations of the included studies as a whole. The next section covers clinical implications regarding RVO2peak and what we suggest as possible future research directions based on the findings we have presented. Finally, we include a one-paragraph conclusion.

**13.** **Subsections:** Please remove the Declarations section (and all subsections) and instead ensure that all the information is captured in the submission form questionnaire in Editorial Manager.

**Response:** These sections have been removed from the manuscript.

**14.** **References:** Please use ‘et al’ after listing the first six authors in your references, and remove italic/bold formatting.

**Response:** All references have been reformatted as requested.

**Reviewer #1**

Thank you for preparing this wonderful manuscript. Due to competing interests I am not able to provide any further recommendations. However, I would like to point out a few things which I find important and I leave to the Editor whether they will share these comments with you.

**1.**	**Comment:** Numerous experts are against meta-analysis of pre-post intervention studies (or within group differences from RCTs). The authors appropriately downgraded the Evidence to Low certainty, however, I still believe that these results shouldn't be central in your manuscript.

**Response:** Thank you for your comment, we have now included the RCTs (interventions relative to controls) as the primary meta-analysis. Given that there were only a small number of RCTs to be included in the meta-regression or compare across subgroups, we have decided to include the original pooled pre-post and RCT exercise interventions as a secondary meta-analysis, of which we have drawn upon significant differences in our discussion. The following excerpt is taken from the Cochrane Handbook (see point i), as rationale for pooling the pre-post and RCT exercise interventions for the meta-regression and subgroup analyses:

“The main drivers that may lead to the inclusion of non-randomized studies include: (i) when randomized trials are unable to address the effects of the intervention on harm and long-term outcomes or in specific populations or settings; or (ii) for interventions that cannot be randomized (e.g. policy change introduced in a single or small number of jurisdictions) (see Chapter 24).”

**2.**	**Comment:** A meta-analysis of RCTs is more appropriate (section 3.2.3. RCTs in your manuscript). You do not discuss high heterogeneity in before-mentioned section (was higher than 80%). I believe that results from RCTs should be presented as primary, with subgroup analysis based on types of interventions/controls used, mean age, or health status of study participants. Meta-regression may be useful as well. Also, I suggest that leave-one-out analysis is implemented.

**Response:** Thank you for your comment. We have now included the RCTs (interventions relative to controls) as the primary meta-analysis and reported heterogeneity in the text. Given the wide range of injury characteristics (time since injury, level of injury and injury severity) of individuals included in this review, one would expect to observe significant heterogeneity and therefore we do not feel that further discussion on this is warranted relative to what was already included in the original submission. Furthermore, as there are a much smaller number of RCTs, in comparison to the pooled meta-analysis of pre-post and RCT exercise interventions, we are not able to perform subgroup analyses as these comparisons would be underpowered. We are also unable to perform a meta-regression on the RCTs only for this same reason. Our rationale to pool study designs can be found above. With regards to age, this could not be included as a subgroup in the pooled meta-analysis given that it is a continuous variable (i.e., there are no specific thresholds to dichotomise age into “young”, “middle-aged” and “older” populations), however we have captured the effect of this variable in the meta-regression in the secondary, pooled analysis. Leave-one-out analyses have now been included too as per the reviewers suggestion.

**3.**	**Comment:** Conclusions are not reflecting the true nature of summarized evidence. In particular, I suggest to implement statements such as: "based on evidence of low certainty..." when summarizing the most important findings.

**Response:** We have added this to the conclusion (Line 1060-1062).

**4.**	**Comment:** Directions for future research should focus on identified literature gaps and methodological issues across studies which were discussed in manuscript.

**Response:** Thank you for your comment. We have already addressed that the dose-response relationship between level of physical activity and CRF is currently the biggest, and most necessary, gap in the literature that needs to be explored. We have also highlighted that poor reporting and lack of pre-registration of trials needs to be improved. A statement at the end of the conclusion has been added to emphasise these points.

**Reviewer #2**

**1.**	**Comment:** I confine my remarks to statistical aspects of this paper. These were very well done and I recommend publication.

**Response:** We thank you for taking the time to review our manuscript and for recommending publication.

**Reviewer #3**

**1.**	**Comment:** In their manuscript entitled "The impact of physical activity and exercise on aerobic capacity in individuals with spinal cord injury: A systematic review with meta-analysis and meta-regression" the authors made analyses on ninety studies and a total of 1,191 participants to conclude that performing exercise >2 weeks results in significant improvements in absolute V̇O2peak, relative V̇O2peak and peak power output (PPO) in individuals with SCI. With caution due to the heterogeneity of the subgroup, the authors also reported variables that could influence the effect of training on the PPO, such as time since injury, level of injury, exercise intensity, or training volume. The meta-analyze is well conduced and the results are correctly and exhaustively reported although figures would have been preferred for the main outcomes.

**Response:** Thank you for your comment. We have included figures for both the new primary meta-analysis (RCTs) (Figure 2) and summary of the pooled pre-post and RCT exercise intervention subgroup pooled effects (Figure 3).

**2.**	**Comment:** Why didn't the authors include study with whole-body exercise training such as FES-row training? (for exemple: Wheeler, Arch Phys Med Rehabil 2002 or Qiu, Medicine and Science in Sports and Exercise 2016). It seems to me that those RCTs are missing and the reasosn for not considering this training form is not explain in the methods.

**Response:** Both Wheeler and Qiu papers are included in the meta-analysis already (see S6). Perhaps the reviewer mistaken these for RCTs. These studies are pre-post exercise interventions.

**3.**	**Comment:** Limitation paragraph: first sentence of Page 28: studies with "more rigorous exercise interventions" already exist (see above). Examples need to be provided and the authors need to explain why they did not include these studies here.

**Response:** Please see our response above regarding the studies you have mentioned. Our GRADE assessment downgraded the certainty of the evidence based on the criteria described in Table 6. Our systematic review included 67 pre-post studies and 29 RCTs, showcasing the relative discrepancy in rigorously designed, Level 1 evidence supporting the benefits of exercise on CRF in this population. Where there was a difference in the pooled effects between study designs, we downgraded the certainty of the evidence. As such, we strongly believe that more rigorous exercise interventions are required to improve the state of the literature and thus our understanding of the effects of physical activity and exercise on aerobic capacity in individuals with SCI.

**4.**	**Comment:** Line 837: please recall in the sentence the number of RCTs vs. pre-post studies

**Response:** These have been added to Line 1006.

**5.**	**Comment:** Third paragraph of limitation: line 847: Multiples comparison analyses increase chances to find a comparison significant. i.e. "subgroup differences for PPO based on TSI, neurological level of injury, exercise modality, exercise intensity, method of exercise intensity prescription, and frequency of sessions". was the p value adjusted for this bias?

**Response:** Thank you for your comment. We can confirm that individual subgroup p-values were adjusted for multiple comparisons via the Bonferroni correction method. The thresholds for statistically significant subgroup differences (i.e., as you’ve questioned: TSI, neurological level of injury etc.) were adjusted for the number of subgroup comparisons. This information was provided in the footnote of Tables 3 and 4 in S6 but is now also detailed in the methods (Line 381-383).

**Reviewer #4**

**1.**	**Title**

**Comment:** Since the authors have included observational studies in their review I think the word impact might be avoided in favour of something more neutral. If the review was restricted to trials then impact would be fine.

**Response:** We thank you for your comment. We have updated our title to “Effect of” in line with PLoS Medicine guidance of reviews including RCTs. Observational studies were included in this review to ensure we had gathered an all encompassing evidence–base of physical activity and exercise following spinal cord injury. These observational studies made up a small percentage of the total number of studies included and only represent one of the secondary meta-analyses (S13). Consequently, we feel the updated title is justified.

**2.**	**Page 7**

**Comment:** The authors themselves state on page 28 that they are aware that restricting the study to English language only is a limitation so why do it?

**Response:** Please see the response to editorial comment #11 above that outlines how we have now screened non-English language studies. Three additional studies have been included as a result.

**3.**	**Page 8**

**Comment:** For reproducibility we need to know which information was supplied and which we cannot find in the primary papers. Acknowledging the people who provided data would be nice too.

**Response:** Authors that provided responses to their study have now been cited in the methods (Line 302). Studies that required data imputation have also been listed in supplementary material (S8) should readers be interested in reproducing our findings.

**4.**	**Page 9**

**Comment:** In fact the correlations are labelled Table e5. These seem very high for pre–post correlations given that they should not exceed the reliability of the measure. The formulas look right though.

**Response:** The correlations can be found in S4 as stated in the manuscript. We can confirm that we consulted and subsequently followed the guidance in the Cochrane Handbook stating “Before imputation is undertaken it is recommended that correlation coefficients are computed for as many studies as possible and compared. If these correlations vary substantially then sensitivity analyses are particularly important.” We can confirm that all correlation factors were similar across study designs, which therefore removed the need for a sensitivity analysis. The formulas were used correctly and thus we are confident that the calculated correlation factors are sound.

**5.**	**Page 9**

**Comment:** The robvis package is properly cited on page 11 but the packages used for the meta–analysis does not appear to be mentioned. From the GitHub repository it seems both metafor and meta were used.

**Response:** This information has now been detailed in the manuscript (Lines 373 and 381).

**6.**	**Page 10**

**Comment:** Deciding to choose the meta–analysis model based on observed heterogeneity is often recommended but these days it is usually preferred to base it on the scientific situation. See Hedges and Vevea (1998) for an early argument and Rice et al. (2018) for a detailed modern version. To quote the latter:

Measures of heterogeneity should not be used to determine whether fixed effects analysis is appropriate, but users should instead make this decision by deciding whether fixed effects analysis — or some variant of it — answers a question that is relevant to the scientific situation at hand.

It is up to the authors to decide which fits their scientific question best.

L V Hedges and J L Vevea. Fixed– and random–effects models in meta– analysis. Psychological Methods, 3:486–504, 1998.

K Rice, J P T Higgins, and T Lumley. A re–evaluation of fixed effect(s) meta–analysis. Journal of the Royal Statistical Society, 181:205–227, 2018.

**Response:** We agree that the decision to choose a meta-analysis model, whether it is a common, fixed or random effects model, should be based on several factors, including the clinical/scientific situation and the characteristics of the included studies. We recognise that for studies with rare events, often the fixed effects model is chosen as a pooling method. Our decision to use a random effects model was based on the expectation that the treatment effects would be similar but not identical. The sample sizes across studies vary and we believe that the pooled population is unrepresentative of the overall SCI population. The included studies have different exercise intervention components, variable participant characteristics (by nature of SCI being a heterogeneous condition depending on the neurological level and severity of injury), and were conducted in at least 21 countries across 5 continents with a myriad of diverse populations. The latter point has now been visualised in Figure 1 of S6. Therefore, a random effects model may better account for the variability in the true effect size across studies. We also had *a priori* concerns about the magnitude of the between-study variability, which explains our decision to use a random effects model.

**7.**	**Page 10**

**Comment:** What funnel plots and regression tests hope to identify is small study effects. Calling it publication bias implies you know the aetiology.

**Response:** Thank you for this comment. Indeed, small study effects refer to the phenomenon where smaller studies in a meta-analysis tend to show larger treatment effects than larger studies, even after accounting for differences in sample size and study quality. This may be due to a variety of factors, such as publication bias, heterogeneity, or chance. Small study effects can potentially bias the overall meta-analysis estimate towards a more positive effect size.

Publication bias, on the other hand, refers to the selective publication of studies based on their results. This can occur when studies with positive or statistically significant results are more likely to be published than studies with null or negative results. This can lead to an overestimation of the overall effect size in a meta-analysis if studies with null or negative results are not included.

We agree that we do not know the aetiology, and have hence changed the wording to small study effects.

**8.**	**Page 15**

**Comment:** It is hard to know what sense to make of the findings on what the authors term imputed data. Reading data off a graph should not produce a systematic bias so does this imply conversion from medians is biased?

**Response:** Whilst extrapolating data from figures in Adobe, as well as converting medians and interquartile ranges into means and standard deviations (as per Cochrane guidance), is not expected to produce any unnecessary systematic bias, we felt that it was important to be transparent with this approach given that we were not including original means and standard deviations. To be completely transparent with both of these approaches, we have now reported the pooled effects for studies with imputed data from conversions, extrapolated data from figures, and data extracted directly from papers. We have now listed these categorised studies in supplementary material (S8) should readers wish to identify these.

**9.**	**Page 15**

**Comment:** I think that in the moderator analysis the authors need to be more cautious. These are ecological effects so, for instance, it is not age which leads to increase in the CRF outcomes but being enrolled in a study with a higher average age. Although related these are not the same.

**Response:** Thank you for your comment. We have made an alteration to this statement in the abstract (Line 92-93) and the results in the manuscript (Line 573-577).

**10.** **Page 16**

**Comment:** I agree the funnel plots should not provoke any anxiety although in e4 and e6 the outlier spoils the visual impact. That study is probably unlikely to affect the fit of the regression line given its position although it is potentially a high leverage point.

**Response:** Thank you for commenting on this. To ensure that this outlier does not affect the fit of the regression line we have removed this study and performed the Egger’s test again. However, this does not considerably change the outcome for AVO2peak (with study included: z = -1.20, p = 0.23; with study excluded: z = -1.14, p = 0.25) nor PPO (with study included: z = 0.76, p = 0.45; with study excluded: z = 0.81, p = 0.42).

**11.** **Page 17**

**Comment:** Most people would hold that RCTs provide a better assessment of the effect of interventions so I wonder why the analysis of RCTs is treated as a sensitivity analysis rather than as the main analysis?

**Response:** The RCT interventions relative to controls meta-analysis was not treated as a sensitivity analysis. This meta-analysis was included to support the decision to pool the pre-post and RCT exercise interventions together. However, we have now revised our manuscript to include the RCTs as the primary meta-analysis, with the pooled analysis as secondary.

**12.** **Page 21**

**Comment:** Saying the first is a hostage to fortune, ‘to our knowledge’ perhaps safer.

**Response:** We thank you for your comment on this and are very much in agreement with you. We have reworded this in the text (Line 1035).

**13.** **Subgroups**

**Comment:** The subgroup analysis in Tables 3 and 4 includes categories for mixed and not reported. This seems to me hard to justify. Mixed is a mixture in unknown proportions of the two clearly defined categories and not reported may be a mixture or it may be purely one category. I suggest that for the subgroups the analysis is restricted to just those which can be unambiguously allocated.

**Response:** We thank you for your comment. We took this approach to be transparent and include all studies given that the pooled effects would change with the removal of mixed and not reported categories. For example, if a study included a range of neurological levels of injury (i.e., paraplegic and tetraplegic participants) it was classed as mixed, yet these participants may have all had a motor-complete SCI. This means that if this study was removed from the mixed category for the “neurological level of injury” subgroup analysis, but remained for the “injury severity” subgroup analysis, the pooled effects across both analyses would not be consistent. Furthermore, we are transparent with the studies included in the mixed categories. Tables 1-2 of supplementary material for the secondary, pooled meta-analysis (S6) provides proportions of participants included in each mixed category (i.e., % acute for time since injury, % para for neurological level of injury, % motor-incomplete for injury severity). Readers are also able to identify the studies included in these mixed categories in the individual study descriptions table and the forest plots in this supplementary file.

Additionally, one of the take-home messages of our review is that there is poor reporting across studies with regards to injury characteristics of the included participants as well as the exercise intervention parameters prescribed across studies. We feel that it is important to include the NR/CD categories to highlight how poor reporting may lead to effects that are not aligned with the pooled effect. Some NR/CD categories have subgroup effects that are either attenuated or inflated in comparison to other categories. For example, the NR/CD subgroup effect based on “frequency of sessions” for RVO2peak is 0.9 mL/kg/min, yet the other subgroups that include studies reporting sessions per week of <3, ≥3 to <5 and ≥5 observe subgroup effects of 2.9, 2.8 and 3.5 mL/kg/min, respectively. As such, you can see how poor reporting may underestimate the true effect.

**14.** **Multiplicity**

**Comment:** We can see in various places, for instance Figures e1 to e3 in supplementary material S4, that some studies are repeated. This violates one of the assumptions of meta–analysis that the effect sizes are independent.

**Response:** We took a rigorous approach to ensure that all exercise interventions were 'independent', regardless of whether multiple interventions were from one study. Where there was a crossover of participants from one intervention arm to another, we only included studies with a substantial washout period before participants performed the subsequent intervention. Had we not taken this approach and excluded multiple intervention arms from specific studies, we would not have been able to present an accurate assessment of the literature. A similar approach has been taken in a recent systematic review with meta-analysis on the effects of aerobic exercise intensity on cardiorespiratory fitness in individuals undergoing cardiac rehabilitation, published in the British Journal of Sports Medicine (Mitchell et al., 2018). We have made it clear in the supplementary material the trials that have multiple arms and how they have been reported in the analyses, to be transparent about the assumption of independence of effect sizes.

Mitchell BL, Lock MJ, Davison K, et al. What is the effect of aerobic exercise intensity on cardiorespiratory fitness in those undergoing cardiac rehabilitation? A systematic review with meta-analysis. British Journal of Sports Medicine 2019;53:1341-1351.

**15.** **Language restriction**

**Comment:** Various empirical studies have indicated that the main bibliographic databases have varied and incomplete coverage of material not in English (Pilkington et al., 2005; Shenderovich et al., 2016), and have limited coverage of material from low and middle–income country journals (Kieling et al., 2009; Syed Sheriff et al., 2008). Despite that the authors’ search does appear to have turned up some relevant studies so extending to databases with coverage of other literatures might be helpful. WHO sponsors some. In the event of all the authors being monoglot English speakers they might be interested in an empirical study of machine translation in reviews (Jackson et al., 2019). There has been a study in this journal which went to extraordinary lengths to obtain full world-wide coverage (James et al., 2022).

J L Jackson, A Kuriyama, A Anton, A Choi, J-P Fournier, A-K Geier, F Jacquerioz, D Kogan, and R Sun. The accuracy of Google Translate for abstracting data from non–English–language trials for systematic reviews. Annals of Internal Medicine, 171:677–679, 2019.

T James, N Mukadam, A Sommerlad, H R Pour, M Knowles, I Azocar, and G Livingston. Protection against discrimination in national dementia guideline recommendations: A systematic review. PLOS Medicine, 19 (e1003860), 2022.

C Kieling, H Herrman, V Patel, and J J Mari. Indexation of psychiatric journals from low– and middle–income countries: a survey and a case study. World Psychiatry, 8:40–44, 2009.

K Pilkington, A Boshnakova, M Clarke, and J Richardson. ‘No language restrictions’ in database searches: what does this really mean? The Journal of Alternative and Complementary Medicine, 11:205–207, 2005.

Y Shenderovich, M Eisner, C Mikton, F Gardner, J Liu, and J Murray. Methods for conducting systematic reviews of risk factors in low– and middle–income countries. BMC Medical Research Methodology, 16(32), 2016.

R J Syed Sheriff, C E Adams, P Tharyan, M Jayaram, L Duley, and the PRACTIHC Mental Health Group. Randomised trials relevant to mental health conducted in low and middle–income countries: a survey. BMC Psychiatry, 8(69), 2008.

**Response:** Please see previous response to editorial comment #11 regarding non-English language studies.

**Reviewer #5**

**1.**	**Comment:** The second paragraph of the introduction can be appropriately shortened and the parts that are not related to the research can be deleted. You can consider merging it with the third paragraph.

**Response:** We thank you for your comment on the introduction. We agree that this could be shortened and have removed background on the influence of neurological level of injury on sympathetic control and the resulting reduction in exercise capacity. The references [15,16] that were originally used to inform the introduction have been moved to the discussion to support our comment on the influence of neurological level of injury on changes in peak power output (Line 740).

**2.**	**Comment:** The exclusion criteria in the method must be rewritten, as the exclusion criteria are supplementary explanations to the inclusion criteria, rather than the opposite of the inclusion criteria.

**Response:** Thank you for your comment. Whilst we understand that it may be useful to have exclusion criteria that completely oppose the inclusion criteria, we feel that the exclusion criteria in its current form is appropriate. Furthermore, to the best of our knowledge Cochrane guidance on systematic reviews does not specify that exclusion criteria should be the opposite of the inclusion criteria. Therefore, this remains unchanged (except for the removal of non-English as an exclusion criterion). This would also require us to re-screen 1,926 full texts to re-classify their reason for exclusion based on merely being opposite to the inclusion criteria, which seems unnecessary.

**3.**	**Comment:** Adverse event events are a key focus of attention, and can the results be presented in a chart or graph?

**Response:** Thank you for your comment. We have summarised these in a figure which is included in supplementary material (S7). Given the number of tables and figures already included in the manuscript it was decided that this figure would be best placed in supplementary material.

**4.**	**Comment:** The main effect was demonstrated in Tables, why not use a forest plot instead, which will look more intuitive?

**Response:** Thank you for your comment. Whilst we agree that forest plots are a common way to present pooled effects for outcomes in meta-analyses, given the number of exercise interventions in our study we concluded that Forest plots would be too large to include within the manuscript. Furthermore, given that we categorised by subgroups based on injury characteristics and exercise intervention parameters, 27 forest plots were generated in total for the pooled secondary meta-analysis that compared across subgroups (nine subgroup analyses across AVO2peak, RVO2peak and PPO) and thus would not be realistic to include within the main manuscript itself. However, forest plots for all of our meta-analyses are included in the supplementary material, with statements in the text directing readers to the specific files for each analysis. We have now summarised the subgroup effects for RVO2peak and PPO in Figure 3, which we hope the reviewer will find more intuitive. Also, we have now included an adapted Forest plot for the primary (RCT) meta-analysis, which we feel is an appropriate way to show these data for the smaller proportion of RCTs.

**5.**	**Comment:** Table 6 is too large, so some content can be simplified to make the layout look better.

**Response:** Thank you for your comment. We have taken this suggestion on board and have condensed the table by trimming information from the comments and RoB sections. We have also removed other tables from the manuscript to allow for including more intuitive visualisations of the compiled data. Rather than include our justifications in the written text of the manuscript (which is already comprehensive) for either downgrading or not downgrading, we felt that summarising this information in the GRADE table is the most appropriate way to include/present this information.

**6.**	**Comment:** This study only mentions CRF as the outcome indicator, why not pay attention to the changes of other functional indicators related to spinal cord injury, such as ASIA grading?

**Response:** Thank you for your comment. Whilst this would indeed be an interesting research question, the aim of our review was to focus on cardiorespiratory fitness (CRF) outcomes only. Previous research has demonstrated that CRF is a modifiable risk factor that is perhaps more important than other traditional risk factors such as obesity, hypertension, smoking etc. and is strongly associated with mortality (see our introduction). Indeed, a recent paper has advocated that guideline bodies should consider including CRF in standard risk panels for mortality risk assessment (Laukkanen et al., 2022). Thus, we sought to pool the existing evidence for changes in CRF following exercise interventions in individuals with spinal cord injury. In our secondary, pooled meta-analysis we subgrouped exercise interventions by motor-complete (ASIA A/B) and motor-incomplete (ASIA C/D) to explore how injury severity could influence responses to an exercise intervention. Furthermore, it is uncommon for exercise interventions to assess change in ASIA grade; instead, the ASIA grade is usually reported at baseline to provide participant demographic information. Studies reporting these changes are normally limited to rehabilitation studies, e.g., locomotor/gait training modalities or other activity-based therapy interventions rather than volitional exercise per se, as shown in a recent systematic review with meta-analysis investigating the clinical benefits of rehabilitation training in SCI (Duan et al., 2021). Only 3 out of 30 included studies reported pre-post ASIA motor score and class or ASIA upper/lower extremities motor score (ASIA UEMS/LEMS).

Ruimeng, D., Mingjia, Q., Yashuai, Y., Miaoman, L., Tao, L., Wei, H., Junxiao, G., Meng, Z., Xiaobing, Y. 2021. Clinical Benefit of Rehabilitation Training in Spinal Cord Injury: A Systematic Review and Meta-Analysis. SPINE, 46(6):E398-E410.

Laukkanen JA., Isiozor NM., Kunutsor SK. 2022. Objectively Assessed Cardiorespiratory Fitness and All-Cause Mortality Risk: An Updated Meta-analysis of 37 Cohort Studies Involving 2,258,029 Participants. Mayo Clinic Proceedings, 97(6):1054-1073.

**7.**	**Comment:** How the outcome measures (AV̇O2peak) or relative V̇O2peak (RV̇O2peak) are defined? What is the difference between V̇O2peak and AV̇O2peak/ RV̇O2peak?

**Response:** These were defined in the methods section of the manuscript (Line 252-254). Both absolute peak oxygen uptake (AVO2peak, quantified as L/min) and relative peak oxygen uptake (RVO2peak, quantified as mL/kg/min) are common terms used in the field of exercise physiology.

**8.**	**Comment:** In the conclusion, the author mentioned "prescribed via a percentage of oxygen consumption or heart rate", What are the specific values?

**Response:** Thank you for your comment. In the conclusion we have now included the minimum percentages required for prescribing exercise intensity via oxygen consumption or heart rate methods. These percentages meet the “moderate” exercise intensity classification, as defined by the American College of Sports Medicine (ACSM) exercise intensity thresholds. These thresholds are clearly indicated to readers in the supplementary file. Given we identified that improvements in PPO were greater with exercise prescribed at a moderate-to-vigorous-intensity, we have now specified the minimum moderate-intensity thresholds in this sentence.

**9.**	**Comment:** The final conclusion should recommend which type of exercise, intensity, and duration should be clarified.

**Response:** In our pooled, secondary meta-analysis, whilst there was a subgroup difference for RVO2peak based on length of intervention following the updated search (Line 1067-1068), the majority of subgroup differences were for PPO. In the conclusion we have recommended what exercise modality, intensity, method of intensity prescription and frequency should be used to yield optimal changes in PPO. We did not subgroup by duration in our analysis as this variable is a product of volume (e.g., frequency x duration); yet this had no significant subgroup differences for PPO and was therefore not included in the conclusion.

**Reviewer #6**

**1.**	**Comment:** This review will be a strong contribution to the literature as it stands. However, according to the Cochrane guidelines, it is recommended that the authors rerun or update the search within 12 months prior to publication.

**Response:** Thank you for this comment. The search has been updated to include studies published between 18th July 2021 and 25th March 2023. An additional 8 studies have been included from this period.

**2.**	**Line 74-76**

**Comment:** Suggestion to revise the statement to include what there is a significant improvement from? For example, add to the end of the statement: “in all 90 studies after an exercise intervention”.

**Response:** We have now specifically stated that these improvements were following exercise interventions >2 weeks (Line 73 and 87).

**3.**	**Line 86-88**

**Comment:** Great summary statement. It is unclear why this final conclusion statement only focuses on power output.

**Response:** Thank you for your comment. As there were no subgroup differences for AVO2peak these were not included. However, for RVO2peak we have now included that exercise interventions up to 12 weeks exhibit greater changes than interventions >12 weeks. This was a new finding following the updated search and re-analysis. Please see Line 88-90.

**4.**	**Line 105**

**Comment:** Consider adding the word “factors” between “confounding and”.

**Response:** This line has been removed due to the inclusion of an ‘Author Summary’ rather than ‘Key Points’.

**5.**	**Line 130**

**Comment:** Consider removing the second mention of “cardiorespiratory fitness” and replace with CRF. The acronym was mentioned in Line 129.

**Response:** Thank you for spotting this, we have made this change in the manuscript (Line 180).

**6.**	**Line 176**

**Comment:** Consider revising to “…and not other outcomes in individuals with SCI.”

**Response:** We have made this change in the manuscript (Line 218).

**7.**	**Line 430**

**Comment:** Were adverse events defined as events causing cessation of exercise or reported after the exercise intervention? Consider defining as serious adverse events for events that caused cessation of the exercise session or exercise intervention and adverse events for those that were reported after the session or intervention.

**Response:** Thank you for this great suggestion, this is something that all exercise intervention studies should look to report going forward. However, for this review we were unable to dichotomise these into serious adverse events (SAEs) and adverse events (AEs) due to poor reporting across studies. Furthermore, according to the National Institute of Health the definition of a SAE is “any adverse event that:

-	Results in death

-	Is life threatening, or places the participant at immediate risk of death from the event as it occurred

-	Requires or prolongs hospitalization

-	Causes persistent or significant disability or incapacity

-	Results in congenital anomalies or birth defects

-	Is another condition which investigators judge to represent significant hazards”

As studies did not provide extensive detail on the nature of their adverse events, or did not provide the specific number of participants experiencing each adverse event, we were unable to categorise these as such. Furthermore, we attempted to provide a summary of adverse events that were either related or unrelated to the exercise intervention but were, again, unable to due to poor reporting. We have now included a sentence in the discussion that proposes a standardised procedure for AE reporting in the future (Line 946-949).

**8.**	**Line 435-440**

**Comment:** These quotes are helpful to inform the nature of the adverse events. Are the quotes presented the most extreme adverse events? Consider describing how these quotes were chosen for Section 3.1.3.

**Response:** We categorised adverse events into subgroups based on 1) bone, joint or muscular pain, 2) autonomic or cardiovascular function, 3) skin irritation or pressure sores, and 4) other. For each of these categories we provided the number of participants that had experienced an adverse event. In four pre-post studies the authors did not specify the number of participants for each adverse event, which made it impossible to include these numbers in the totals for each category. Therefore to ensure these were not missed in the review we included quotes to highlight the adverse events in these specific studies and helped to support our discussion point on the poor reporting of adverse events within the literature. We have clarified this in the text (Line 518-519).

**9.**	**Line 584-585**

**Comment:** Consider revising to clarify this statement. Are the authors suggesting that exercise intervention studies conducted in the inpatient rehabilitation phase are providing concurrent rehabilitation (i.e., a greater dose of exercise per week due to study involvement)?

**Response:** We thank you for your comment. We have made this clearer in the text (Line 699-709).

**10.** **Line 602-603**

**Comment:** It is unclear why there is a W after 16 and 9 in this sentence.

**Response:** The unit for peak power output (PPO) is Watts, abbreviated as W. This was defined earlier on in the manuscript (Line 300).

**11.** **Line 715-717**

**Comment:** This section on exercise intensity is thoughtfully written. Consider adding an additional sentence to provide further direction on how to corroborate safety and feasibility of high intensity interval training (HIIT). Alternatively, perhaps there is a recommendation for the style of HIIT protocol to prescribe.

**Response:** Thank you for your comment on this section. We have clarified the different exercise modalities included in the current review as well as suggested how future research could explore the use of more “real-world” strategies (i.e., virtual HIIT), to account for individuals with barriers to participating in physical activity and exercise sessions outside of their own home. As HIIT is still a vastly under-researched exercise strategy in the SCI population specifically, we are unable to provide concrete recommendations on the ideal protocol to prescribe and so have called for more research into this area. However, we have provided guidance on how to monitor participant safety during high-intensity exercise. Please see updated text on Lines 858-872.

**Additional Notes/Changes**

Please note, there are no tracked changes in the supplementary files given that the majority of tables/figures were updated.

Based on the feedback from the reviewers and changes in our findings following the updated search, we have included the following major additions to the manuscript:

1. There is now a subgroup difference based on length of intervention for RVO2peak. This is described in the abstract (Line 88-90), results (Line 535-536), conclusion (Line 1067-1068) and is explored in the discussion (Line 931-937). Individual subgroup effects are reported in Figure 3.
2. There is now a subgroup difference based on exercise modality for RVO2peak. This is described in the abstract (Line 88-90), results (Line 535-536), conclusion (Line 1062-1063) and is explored in the discussion (Line 797-798). Individual subgroup effects are reported in Figure 3.
3. Findings from the meta-regression reveal that mean age is associated with AVO2peak and RVO2peak changes following exercise interventions. There are no longer any associations between PPO and both exercise volume and method of exercise intensity prescription. This has been updated in the abstract (Line 92-93 and 105), results (Line 571-585) and conclusion (Line 1068-1069). We have included some thoughts on these findings in the discussion too (Line 772-791).
4. The GRADE certainty rating for RVO2peak has changed from Low to Moderate (Table 2). Given that we performed a sensitivity analysis on studies with low and high risk of bias and observed no differences, we can justify not downgrading each CRF outcome based on this criterion.
5. We have now included an additional 3 figures. Figure 2 visualises the participant demographics, exercise intervention parameters, and pre-post CRFvalues for both control and intervention groups of the RCTsincluded in the primary meta-analysis. Figure 3 summarises the subgroup effects and differences for the secondary, pooled meta-analysis of independent pre-post and RCT exercise interventions. These were reviewer suggestions to visually summarise the data rather than include the summary statistics tables included in the original submission. We have also included firepower plots for each CRF outcome in Figure 4. This was not included in the original submission given that the manuscript supporting the metameta R package had not been published in a peer-reviewed journal. This figure seeks to visually present the study-level statistical power of the summary effect size and a range of hypothetical effect sizes for each CRF outcome. Within each outcome, we have separated into pre-post exercise interventions and RCT studies to provide a comparison between the two study designs included in the secondary, pooled meta-analysis. Given that the nature of RCTs are to provide a more rigorously designed study to compare the effect of an intervention relative to a control group control group than non-randomised study designs without a control group without a control group, it is perhaps perhaps not surprising that the observed effect sizes and subsequent statistical power across CRF outcomes are greater in this study design in comparison to the pre-post studies. Study-level statistical power is rarely reported in meta-analyses and so we believe this provides an informative addition to the manuscript.
6. The risk of bias assessments section in the methods has been moved to before the data synthesis and analysis section (Line 306-316). We felt that as risk of bias was included as a moderator in the meta-regression, it would be better placed prior to this so that the reader was aware of the risk of bias assessment tools utilised. so that the reader was aware of the risk of bias assessment tools utilised.
