# Supplementary material for: Exercise and aerobic capacity in individuals with spinal cord injury: A systematic review with meta-analysis and meta-regression
Source: PLoS Med. 2023 Nov 27;20(11):e1004082. doi: 10.1371/journal.pmed.1004082 (PMC10712898; doi:10.1371/journal.pmed.1004082)
Supplement: Attachment — Submitted filename: Rebuttals Hodgkiss et al PLoS Med October.docx [file pmed.1004082.s018.docx]

We thank the Editors and the Reviewers for their comments and suggestions. We have addressed these point-by-point below.

**Editorial Comments**

1. **The editorial team feels strongly that several dimensions remain to be resolved:**

**RTCs (only), RTCs+ pre-post studies, RTCs + everything - uncontrolled and after study effect size. Please systematically conduct the analysis for each of the subgroup analyses crossed with study type.**

**Response:** We thank the Editors for their comment. Our review presents the RCTs (exercise intervention vs control groups) as the primary meta-analysis given this is the most robust, high quality and precise study design that can reliably estimate causation. Following Reviewer 4’s comment regarding the need to exclude studies with “mixed, not reported or cannot determine” injury characteristics or exercise intervention parameters, there were not enough RCTs left to perform subgroup analyses on this study design alone. Cochrane provides the following guidance on subgroup analyses:

*“It is very unlikely that an investigation of heterogeneity will produce useful findings unless there is a substantial number of studies. Typical advice for undertaking simple regression analyses: that at least ten observations (i.e., ten studies in a meta-analysis) should be available for each characteristic modelled. However, even this will be too few when the covariates are unevenly distributed across studies.”*

For context, across the 27 subgroup analyses (9 subgroup analyses per cardiorespiratory fitness outcome), only 15 had a sufficient number of studies to run an analysis on RCTs only. Even then, the Cochrane handbook states that more studies would be required in instances where there is substantial heterogeneity, which is the case in the SCI population and as demonstrated in our review (please see I^2^ statistics on Line 466).

Therefore, to be able to perform subgroup analyses, as well as a meta-regression, we included a meta-analysis that pools independent exercise interventions from both RCTs and pre-post studies. A review by Shrier et al. (2007) discussed the inclusion of observational studies in addition to RCTs in meta-analyses. The authors stated *“We conclude that the totality of the evidence suggests that, under some conditions, including observational studies would increase precision appropriately and may produce equally or more relevant and valid results for the question being asked. Doing so could reduce false inferences based solely on RCTs or the inability to make any inferences because of the paucity of RCTs on the subject.”* There is a clear lack of robust, adequately powered RCTs in the field of exercise following spinal cord injury that have assessed each CRF outcome individually and so we felt it was absolutely necessary that other study designs were considered in order to evaluate the state of the evidence at the current time.

Furthermore, we have now re-worded our “secondary meta-analyses” to “additional analyses”. Given the relatively low number of studies included within each study design here [cohort, longitudinal observational, RCT exercise intensity comparisons (i.e., no control groups, two exercise intensities compared with one another)], we have not performed any subgroup analyses on these study designs specifically. We believe that including the results of these analyses inform important discussion points (see below) and we hope you agree that they should not be included as supplementary material.

*Cohort analyses:* There are greater cardiorespiratory fitness (CRF) benefits observed in those individuals who have regularly participated in physical activity and/or exercise over a prolonged period of time. Therefore, perhaps performing greater volumes of exercise per week overtime may lead to a greater CRF and overall improvements in health.

*Cross-sectional associations:* Similarly, individuals who perform greater volumes and intensity of physical activity are associated with a higher CRF, in comparison to individuals who only perform light-intensity activity.

*Observational/longitudinal:* There are significant changes in peak power output, but not absolute or relative peak oxygen consumption, following a period of inpatient rehabilitation. Therefore, more work needs to be done to target improvements in these outcomes in the subacute period following a SCI.

*Exercise intensity comparisons from RCTs:* We revealed that there are no significant differences between moderate- and vigorous-intensity exercise interventions. This is important as it could suggest that shorter exercise protocols such as high-intensity interval training (HIIT) could be as effective as continuous moderate-intensity protocols. Previous work in individuals with SCI has demonstrated that participants found HIIT more enjoyable than moderate-intensity exercise (Astorino and Thum, 2018) and we therefore felt it was important to refer to this in our discussion.

Astorino TA, Thum JS. Within-session responses to high-intensity interval training in spinal cord injury. Disabil Rehabil. 2018;40: 444–449.

Shrier I, Boivin JF, Steele RJ, Platt RW, Furlan A, Kakuma R, Brophy J, Rossignol M. Should meta-analyses of interventions include observational studies in addition to randomized controlled trials? A critical examination of underlying principles. Am J Epidemiol. 2007 Nov 15;166(10):1203-9.

2. **Clarification of the target populations.**

**Response:** Unfortunately, we are not certain what is meant by this comment and have reached out to the Editors to provide further clarification. In our manuscript we have already described that only studies with adults (aged ≥18 years) with an acquired SCI (traumatic, infection, cancer) were included (Lines 249-250). In our introduction we have highlighted how there is a need to explore differences in cardiorespiratory fitness responses between injury-specific characteristics (tetraplegic vs. paraplegic, acute vs. chronic time since injury etc.) (Lines 188-227) and have subsequently described what these subgroups are in the methods section (e.g., acute injury is <1-year, chronic injury is ≥1-year etc.) (Lines 365-377).

3. **Clarification of exposures**

**Response:** Unfortunately, we are not certain what is meant by this comment and have reached out to the Editors to provide further clarification. In our manuscript we have already described that we only included studies with exercise interventions lasting longer than 2 weeks for the primary meta-analysis of RCTs and the pooled meta-analysis of RCTs and pre-post studies (Lines 251-252). We have also stated that we included any studies reporting a measurable exposure variable within cross-sectional studies (i.e., cohort: athletes vs. non-athletes or sedentary vs. active participants; and associations: self-reported or objectively measured habitual physical activity levels) (Lines 252-254).

4. **Clarify study quality (mixing of GRADE evidence)**

**Response:** We believe that our GRADE assessment adhered to guidance provided in the GRADE handbook, whereby RCTs and pre-post (i.e., observational) studies can be considered together in an assessment. For each CRF outcome, we compared the weighted mean differences (WMDs) between the RCTs (interventions relative to control groups) and the pre-post studies. We downgraded the evidence if there was a notable difference across study designs. AVO_2peak_ was downgraded as the WMDs were different (RCTs: 0.16 L/min & pre-post studies: 0.23 L/min), yet they were identical or relatively similar for RVO_2peak_ (RCTs: 2.9 mL/kg/min & pre-post studies: 2.9 mL/kg/min) and PPO (RCTs: 9W & pre-post studies: 11W).

Our approach is similar to another systematic review in the field of exercise in spinal cord injury, whereby Van der Scheer et al. (2017) included RCTs and pre-post studies in their assessments of changes in CRF for 1.) any adult with a chronic SCI, and 2.) young and middle-aged adults with a chronic SCI. They stated that there was ‘moderate confidence in the evidence showing that exercise can improve cardiorespiratory fitness of any adult with chronic SCI’ as well as ‘high confidence in the evidence showing that exercise can improve cardiorespiratory fitness of young and middle-aged adults with chronic SCI’. As both of these assessments included RCTs and pre-post study designs, our rationale appears sound.

van der Scheer JW, Martin Ginis KA, Ditor DS, Goosey-Tolfrey VL, Hicks AL, West CR, Wolfe DL. Effects of exercise on fitness and health of adults with spinal cord injury: A systematic review. Neurology. 2017 Aug 15;89(7):736-745.

5. **Please conduct meta-analysis in a subsequential combination. Regress the effect size estimate across all dimensions**

**Response:** Apologies, we are not certain on what the wording in this comment means exactly. If this refers to a leave-one-out analysis whereby the meta-analysis is conducted sequentially with leaving out one intervention at a time, then we have already presented this for the pooled meta-analysis and can be found in the supplementary material (S8 Sensitivity Analyses) and is reported in the manuscript (Line 569). We have now also performed this leave-one-out analysis for the primary meta-analysis of RCTs, which has been added to the supplementary material.

If the Editors are suggesting that we perform a meta-regression for each of the study designs (i.e., RCTs alone), then unfortunately there are not enough studies to do so. Please see our response to Comment 1.

6. **Please also provide strong rationale why a certain analysis/estimator is potentially elevated and to be viewed as primary analyses.**

**Response:** Does this refer to us presenting the RCTs (intervention vs control groups) as the primary meta-analysis? This study design was chosen to be the primary meta-analysis given these studies are robust, high quality and precise and can reliably estimate causation. We have provided rationale for only performing subgroup analyses on the pooled analysis (RCTs + pre-post studies) as there are a lack of RCTs alone on which to do this. Please see our response to Comment 1.

**Reviewer #4**

1. **I still disagree with the authors about the desirability of including not reported in the analysis of subgroups. I was not suggesting that they should not be reported but I do not see that analysing them with the other groups is helpful. It is mixing two different concepts, the difference between tetraplegic and paraplegic (for example) and the difference between trialists who report the breakdown and those who do not. Including mixed just muddies the waters further.**

**Response:** We thank you for your comment. We have now taken this on-board and have removed the mixed, not reported and cannot determine studies from the subgroup analyses. These summary statistics on these studies for each subgroup analysis have been presented in table format in supplementary material 6. Furthermore, we would like to confirm that any injury characteristics or exercise intervention parameters that were categorised as not reported or cannot determine were coded as missing in the meta-regression and were therefore not included as another comparison. We included mixed where we could include the numbers of tetraplegic and paraplegic individuals within a study.

**Reviewer #5**

1. **The authors have made a thorough effort to comment and revise regarding to questions raised by the reviewers, and I appreciate the work that has been undertaken. I think it will be clinically beneficial.**

**Response:** We thank you for taking the time to review our manuscript for a second time and for recommending publication.

**Additional Changes**

Figure 2 is a visualisation of participant demographics, injury characteristics, exercise intervention parameters, results and risk of bias for the RCT studies included in the primary meta-analysis. Originally, we had only included pre- and post-intervention means and 95% confidence intervals for each study under the results header. However, given that the RCTs are the primary analysis, we have updated this results section to present the results in a forest plot format. Additional forest plots are presented in the supplementary material.

As before, any changes in the supplementary files are not tracked. Additions to these files include:

- S6 (Pooled meta-analysis exercise interventions): Clarification on the removal of mixed, not reported and cannot determine studies. A table with these effect estimates alone has been included.
