# Supplementary material for: Exercise and aerobic capacity in individuals with spinal cord injury: A systematic review with meta-analysis and meta-regression
Source: PLoS Med. 2023 Nov 27;20(11):e1004082. doi: 10.1371/journal.pmed.1004082 (PMC10712898; doi:10.1371/journal.pmed.1004082)
Supplement: Attachment — Submitted filename: Response to Editors Hodgkiss et al PLoS Med.docx [file pmed.1004082.s019.docx]

We thank the Editors and the Reviewers for their comments and suggestions, and for planning to accept for publication once these have been addressed. We can confirm that these changes have been made. Please see below.

**Editorial Comments**

1. **Please revise your title. "Effect of" should be used only if causality can be inferred, i.e., for an RCT only. Given your study also includes non RTC studies, please revise your title and only use causal language in relation to the part where you use RTCs only.**

**Response:** Thank you for this comment. We have revised our title to remove “Effect of”. Throughout the manuscript, we have attempted to remove any causal language that does not relate to the RCTs specifically.

2. **Thank you for including your PRISMA checklist as an SI. Please ensure your use section and paragraph numbers, rather than page numbers (given that there will be no page numbers in the inline publication).**

**Response:** These changes have been made in the PRISMA checklist. All locations now refer to sections and paragraphs within the manuscript or the supplementary material.

3. **To help us extend the reach of your research, please provide any X (formerly known as Twitter) handle(s) that would be appropriate to tag, including your own, your co-authors’, your institution, funder, or lab. Please provide us with any handles you wish to be included when we post about this paper.**

**Response:** These handles have been provided at the start of the manuscript and in the additional information section within the Editorial Manager portal.

**Reviewer #4**

1. **The authors have addressed my remaining point.**

**Response:** We thank the reviewer for their comments throughout the review process.
